# Pharmacological modulation of the cAMP signaling of two isoforms of melanocortin-3 receptor by melanocortin receptor accessory proteins in the tetrapod *Xenopus laevis*

**DOI:** 10.1530/EC-21-0179

**Published:** 2021-10-22

**Authors:** Ying Xu, Lei Li, Jihong Zheng, Meng Wang, Bopei Jiang, Yue Zhai, Liumei Lu, Cong Zhang, Zhe Kuang, Xiaomei Yang, Li-Na Jin, Gufa Lin, Chao Zhang

**Affiliations:** 1 Translational Medical Center for Stem Cell Therapy and Institute for Regenerative Medicine, Shanghai East Hospital, Shanghai Key Laboratory of Signaling and Disease Research, School of Life Sciences and Technology, Tongji University, Shanghai, China; 2 Department of Hematology, Changzheng Hospital, Naval Medical University, Shanghai, China; 3 Key Laboratory of Spine and Spinal Cord Injury Repair and Regeneration of Ministry of Education, Orthopaedic Department of Tongji Hospital, School of Life Sciences and Technology, Tongji University, Shanghai, China

**Keywords:** *Xenopus laevis*, MRAPs, MC3R, tetrapod

## Abstract

As a member of the seven-transmembrane rhodopsin-like G protein-coupled receptor superfamily, the melanocortin-3 receptor (MC3R) is vital for the regulation of energy homeostasis and rhythms synchronizing in mammals, and its pharmacological effect could be directly influenced by the presence of melanocortin receptor accessory proteins (MRAPs), MRAP1 and MRAP2. The tetrapod amphibian *Xenopus laevis* (xl) retains higher duplicated genome than extant teleosts and serves as an ideal model system for embryonic development and physiological studies. However, the melanocortin system of the *Xenopus laevis* has not yet been thoroughly evaluated. In this work, we performed sequence alignment, phylogenetic tree, and synteny analysis of two xlMC3Rs. Co-immunoprecipitation and immunofluorescence assay further confirmed the co-localization and* in vitro* interaction of xlMC3Rs with xlMRAPs on the plasma membrane. Our results demonstrated that xlMRAP2.L/S could improve α-MSH-stimulated xlMC3Rs signaling and suppress their surface expression. Moreover, xlMC3R.L showed a similar profile on the ligands and surface expression in the presence of xlMRAP1.L. Overall, the distinct pharmacological modulation of xlMC3R.L and xlMC3R.S by dual MRAP2 proteins elucidated the functional consistency of melanocortin system during genomic duplication of tetrapod vertebrates.

## Introduction

The melanocortin receptors (MCRs), a subfamily of the seven-transmembrane class A G protein-coupled receptors, can be selectively regulated by pro-opiomelanocortin (POMC)-derived ligands (i.e. adrenocorticotrophic hormone (ACTH) and α/β/γ/δ-melanocyte-stimulating hormone (MSH)). A logical hypothesis predicts that their ancestral genes occurred and developed throughout the evolution of the chordates, accomplished with two entire genome duplications (1R and 2R), while the topic of whether the fifth paralogous melanocortin receptor appeared after local gene duplication is still debated (1, 2, 3, 4). MC1R–MC5R exhibit different ligand preferences and are functionally segregated during the evolution of the chordates. MC1R plays a critical role in skin pigmentation and inflammation; MC2R participates in steroidogenesis and exclusively binds with ACTH on adrenal cortex cells or interrenal cells; MC3R and MC4R modulate energy homeostasis; MC5R takes part in sebaceous gland secretion (5, 6, 7, 8).

The signaling of MC3R and MC4R can be modulated by MRAP1 and MRAP2, which are small single-pass transmembrane G protein-coupled receptor accessory proteins and express in the brain partly, a site with high expression of MC3R and MC4R. Previous studies showed that MRAP1 or MRAP2 could form a particular anti-parallel homodimer, a special structure in which both surfaces of the plasma membrane includes one N-terminus and one C-terminus of each dimer (9, 10). Both MRAP1 and MRAP2 serve important roles in trafficking the MCRs to the cell surface (11, 12, 13). However, only a few species, such as human (12), mouse (14, 15), chicken (16), channel catfish (17), and *Xenopustropicalis* (18), have examined the pharmacological modulation of MC3R by MRAP or MRAP2 proteins. Remarkably, the regulation of MRAP or MRAP2 on MC3R signaling in *Xenopuslaevis* is still unexplored.

*Xenopuslaevis* is a unique tetraploid amphibian species. As previously reported, ancient polyploidization events including 1R and 2R contribute to the occurrence and evolution of POMC and MCRs. However, whole-genome duplication, benefitting species diversity, is a complicated biological process, and polyploidy is extremely rarer in amniotic vertebrates than in non-amniotic vertebrates (19). In vertebrates, unlike extant teleost, in which over 70% of duplicate genes from genome duplication have been lost since the third duplication, more than half of duplicate genes were retained in *Xenopuslaevis* (20, 21). This allotetraploid Xenopus lineage evolves from two different diploid ancestors related to two current subgenomes, L (long chromosomes) and S (short chromosomes), by hybridization and allotetraploidization 17–18 million years ago, which is younger than the ancient genome duplications in the vertebrate. An interesting influence of allopolyploidization is that the degradation in L chromosomes is lower than the S chromosomes, along with more often preserving, fewer missing genes and a smaller amount of pseudogene formation (22). Because of the asymmetry evolution of two subgenomes, evolution divergence has been detected in two homoeologous copies, leading to the appearance of pseudogenization, subfunctionalization, or neofunctionalization (20, 23, 24, 25). These studies signify that *Xenopuslaevis* serves as a powerful model system of studies for vertebrate development and duplicated gene evolution.

As yet, the interaction of MCRs and MRAPs in African clawed frog has not attracted much attention. A previous study on the peripheral melanocortin signaling showed that MC4R acted as an essential player in limb regeneration of *Xenopuslaevis* by mediating energy homeostasis and reactive oxygen species production (26), whereas there was still a vacancy in the pharmacological function of MCRs modulated by MRAPs in L and S chromosomes, especially MC3R, another MCR involved in energy balance. This research elucidated the evolutionary conservativeness of xlMC3Rs in *Xenopuslaevis* by protein sequence alignment, evolutionary tree, and synteny analysis. We also clarified that the mRNA expression of xlMC3Rs was consistent with the distribution of xlMRAP2s, while xlMRAP.L had some discrepancies. Direct protein interactions between xlMC3Rs and xlMRAPs were confirmed by co-immunoprecipitation (Co-IP) and immunofluorescence assays* in vitro*. xlMRAPs exhibited a great impact on the cAMP production of xlMC3R.L/S stimulated by α-MSH. Additionally, cell surface detection by ELISA assay showed that the increasing ratio of xlMRAP2.L/S lowered the surface expression of xlMC3Rs significantly. Altogether, our results demonstrated similar pharmacological profiles of xlMC3R.L and xlMC3R.S, which could contribute to the study of duplicated gene evolution and functional divergence in MCRs.

## Materials and methods

### Plasmids

NCBI (https://www.ncbi.nlm.nih.gov/) was utilized to search the nucleic acid sequences and amino acid sequences of xlmc3r.L, xlmc3r.S, xlmrap2.S, xlmrap2.L, and xlmrap1.L. The xlmrap1.S was not found and considered a generic loss during genomic duplication. Genes were amplified from the cDNA library of an adult male *Xenopuslaevis,* and all the fragments were ligated into pcDNA3.1 (+) plasmid with or without tag and verified by DNA sequencing (Genewiz, Suzhou, China). Plasmids in luciferase reporter assay had no tag. For Co-IP and cell-surface detection by ELISA, xlMC3Rs carried 3×HA tag and xlMRAPs carried 2×Flag tag both at the N-terminal. For bimolecular fluorescence complementation assay, xlMC3Rs plasmids carried a part of the Venus fluorescent protein at the C terminus, while xlMRAPs plasmids carried the other part of the Venus fluorescent protein N-terminal and 2×Flag at the C-terminal.

### Sequence alignments, phylogenetic trees, and synteny analysis

The amino acid sequences were carried out by ClustalW under default configuration. Evolutionary neighbor-joining trees were generated in MEGA5. The percentage of replicated trees inferred from 1000 replicates was marked on the branches. Based on the number of amino acid substitutions at each locus and after excluding all positions containing gaps and missing data, we calculated the evolutionary distance using Poisson's correction method. Synteny analysis was drawn according to the adjacent genomic regions of elephant shark, zebrafish, two-lined caecilian, *Microcaecilia unicolor*, *Xenopustropicalis*, *Xenopuslaevis*, common wall lizard, chicken, mouse, and human (https://www.ncbi.nlm.nih.gov/genome/).

### RNA extraction and reverse transcription

Tissues were collected from an adult male *Xenopuslaevis* (2 years old) after euthanized with an overdose of MS-222 (Sigma‐Aldrich), following the protocol approved by the Institutional Animal Care and Use Committee of Tongji University. Total RNA was obtained by the TRizol (TIANGEN, Beijing, China) after isopropanol precipitation and ethanol washing. Next, 1 μg total RNA isolated from each fresh tissue was incubated at 42°C for 15 min, 95°C for 3 minutes, and stored in low temperature in a 10-µL reverse transcript mixture after removing genomic DNA in a total of 10 µL gDNA Clean-up mixture by the FastKing RT Kit (with gDNase) (TIANGEN). Then, the cDNA was used for PCR.

### Quantitative polymerase chain reaction

RT-qPCR was conducted in a two-step assay. After the RT, we used the SuperReal PreMix Plus (TIANGEN) to prepare the mixture and LightCycler 96 qPCR instrument (Roche) to run the reaction (a pre-cycling hold for 15 min at 95°C, 40 cycles for 10 s at 95°C, 20 s at 60°C, 20 s at 72°C, one melting cycle for 10 s at 95°C, 60 s at 65°C, 1 s at 97°C). The primers were designed by The Primer3 (https://primer3.ut.ee). The 20-μL reaction mixture contained 10 μL of 2× SuperReal PreMix Plus buffer, 0.4 μL each of 10 μM forward and reverse primers, 8.6 μL of H_2_O, and 1 μL of the 1:15 dilution cDNA. The xlactb.L was a reference gene. qPCR primers are as follows: xlmc3r.L_fw GGTGAACGCCACTCTGGACCC; xlmc3r.L_rev ATCAGCCACGGCCAGGCTG; xlmc3r.S_fw GGTCAACACCACCCTGAACCT; xlmc3r.S_rev GTCGGCCACGGCCAGGCTA; xlactb.L_fw TTCACCACCACAGCCGAAAG; xlactb.L_rev TGTCCGTCAGGCAGCTCATA. Experiments were replicated independently at least three times.

### Cell culture and transfection

High-glucose Dulbecco’s modified Eagle’s medium (DMEM) with 10% (v/v) fetal bovine serum (Gibco) and a cell culture incubator with 95% air and 5% CO_2_ at 37°C was used to culture HEK293T cells. Plasmids were transfected with polyethyleneimine (Polysciences, Warrington, PA, USA). The pcDNA3.1 empty vector was used to balance the transfection system.

### Western blot and co-immunoprecipitation

HEK293T cells co-transfected with 3×HA-xlMC3Rs and 2×Flag-xlMRAPs were lysed in the lysis buffer (Beyotime, Shanghai, China) for 1 h at 4°C. The lysate was pre-cleared, and supernatants were incubated with HA-tag rabbit mAb (CST, Boston, MA, USA) overnight at 4°C. The protein A/G agarose beads were added the next day (Beyotime). Beads were washed and resuspended in loading buffer with β-mercaptoethanol (Sangon, Shanghai, China) and boiled at 95°C for 15 min. Samples were loaded into the SDS/PAGE gel wells, and the PVDF membranes (MilliporeSigma) were available for transferring proteins. Then the membranes were blocked in a blocking buffer (Beyotime) for 15 min. HA-tag rabbit mAb (CST), mouse anti FLAG-tag mAb (ABclonal Biotech, Hubei, China), or FLAG-tag rabbit mAb (CST) were diluted at 1:2000 at 4°C overnight and the secondary horseradish peroxidase (HRP)-conjugated antibody (ABclonal Biotech) at 1:4000 dilution for 2 h at room temperature. We used mouse anti FLAG-tag mAb (ABclonal Biotech) in the reaction of 3×HA-xlMC3R.L co-transfected with 2×Flag-xlMRAPs and 3×HA-xlMC3R.S co-transfected with 2×Flag-xlMRAP1.L. FLAG-tag rabbit mAb (CST) was tried in the reaction of 3×HA-xlMC3R.S co-transfected with 2×Flag-xlMRAP2s. Staining signals were visualized via enhanced chemiluminescence reagents (Beyotime). Images were captured by Amersham Imager 600 (GE Healthcare).

### Bimolecular fluorescence complementation assay

Venus fluorescent protein was split into two parts (VF1 and VF2) for bimolecular fluorescence complementation assay. HEK293T cells were transfected with plasmid containing non-fluorescent fragments and the 2×Flag-tag sequence. A 515-nm laser excited fragments when VF1 and VF2 met each other. HEK293T cells were cultured in plates coated with poly-d-lysine solution (Sangon) in advance and transfected with plasmids the next day. DPBS (Sangon) and 4% (w/v) paraformaldehyde (Sangon) were used for washing and fixing cells, respectively, after 24 h transfection. Cells were treated in permeabilization buffer (PBS with 0.3% (v/v) TWEEN and 5% (v/v) goat serum) before incubation with FLAG-tag rabbit mAb (CST) at 1:2000 dilution overnight. After washing with PBS three times, cells were incubated with goat anti-rabbit IgG (Alexa Fluor 647) (1:1000) (Abcam) for 2 h. Before coverslips were mounted and sealed, Gold Antifade Reagent with DAPI (CST) was used to stain nuclei overnight in dark condition. Fluorescent signals were measured by the Zeiss LSM880 AiryScan Confocal microscope and a 60× oil objective.

### Luciferase reporter assay

The 24-well plates were used to culture HEK293T cells. After 24 h, the transient co-transfections of xlMC3Rs and xlMRAP2s at different ratios of 1:0, 1:1, 1:3, and 1:6 were performed. The DMEM with 0.1% BSA (Sangon) was used to dilute α-MSH (GenScript, Nanjing, China) into six concentrations. Moreover, cells were treated in these reagents for 4 h at 37°C. Cells were incubated with α-MSH (GenScript) at 80% maximal effective concentration (EC_80_) and different concentrations of SHU9119 (GenScript) in a cAMP assay. The signaling was measured by the Dual-Glo Luciferase Assay Reagent (Promega). A Spectramax iD3 Multi-Mode Microplate reader examined luminescence. Experiments were replicated independently at least three times.

### Cell-surface detection by ELISA

To detect the cell surface expression of xlMC3R.L and xlMC3R.S by xlMRAPs, the expression of xlMC3Rs and xlMRAPs on cell membrane was measured by cell surface ELISA. Concisely, cells were cultured in 24-well plates, covered by poly-d-lysine solution (Sangon), and transfected with xlMC3R and xlMRAPs at different ratios of 1:0, 1:1, 1:3, 1:6. 24 h later. DPBS (Sangon) and 4% (w/v) paraformaldehyde (Sangon) were used for washing and fixing cells, respectively. To block nonspecific binding, cells were incubated with 5% (m/v) non-fat milk (Sangon) in DPBS (Sangon) for 1 h. These cells were incubated with HA-tag rabbit mAb (CST) or FLAG-tag rabbit mAb (diluted 1:4000) (CST) in DPBS (Sangon) supplemented with 5% (m/v) non-fat milk (Sangon) for 2 h, washed with DPBS (Sangon), and incubated with the secondary HRP-conjugated antibody (1:7500) (ABclonal Biotech) for 1 h. The tetramethylbenzidine (TMB) solution (Beyotime) was added to the plate for 30 min. The reaction was stopped by adding 2 M H_2_SO_4_, and optical density was detected at 450 nm by a Spectramax iD3 Multi-Mode Microplate reader. Experiments were replicated independently at least three times.

### Statistical analysis

The data from RT-qPCR, luciferase reporter assay, and cell surface ELISA were analyzed by GraphPad Prism 6 (https://www.graphpad.com/). Differences of multiple experimental and control groups were analyzed by one-way ANOVA with Tukey’s post hoc test, and two independent groups were compared by the Student’s *t*-test. The tests were performed with a significance level of 0.05. Not significant [ns], *P < 0.05, **P < 0.01, ***P < 0.001, ****P < 0.0001. Data were plotted as mean ± s.e.m. All experiments were repeated at least three separate times.

## Results

### Conserved evolution and expression of xlmc3r.L and xlmc3r.S

**Figure 1:**
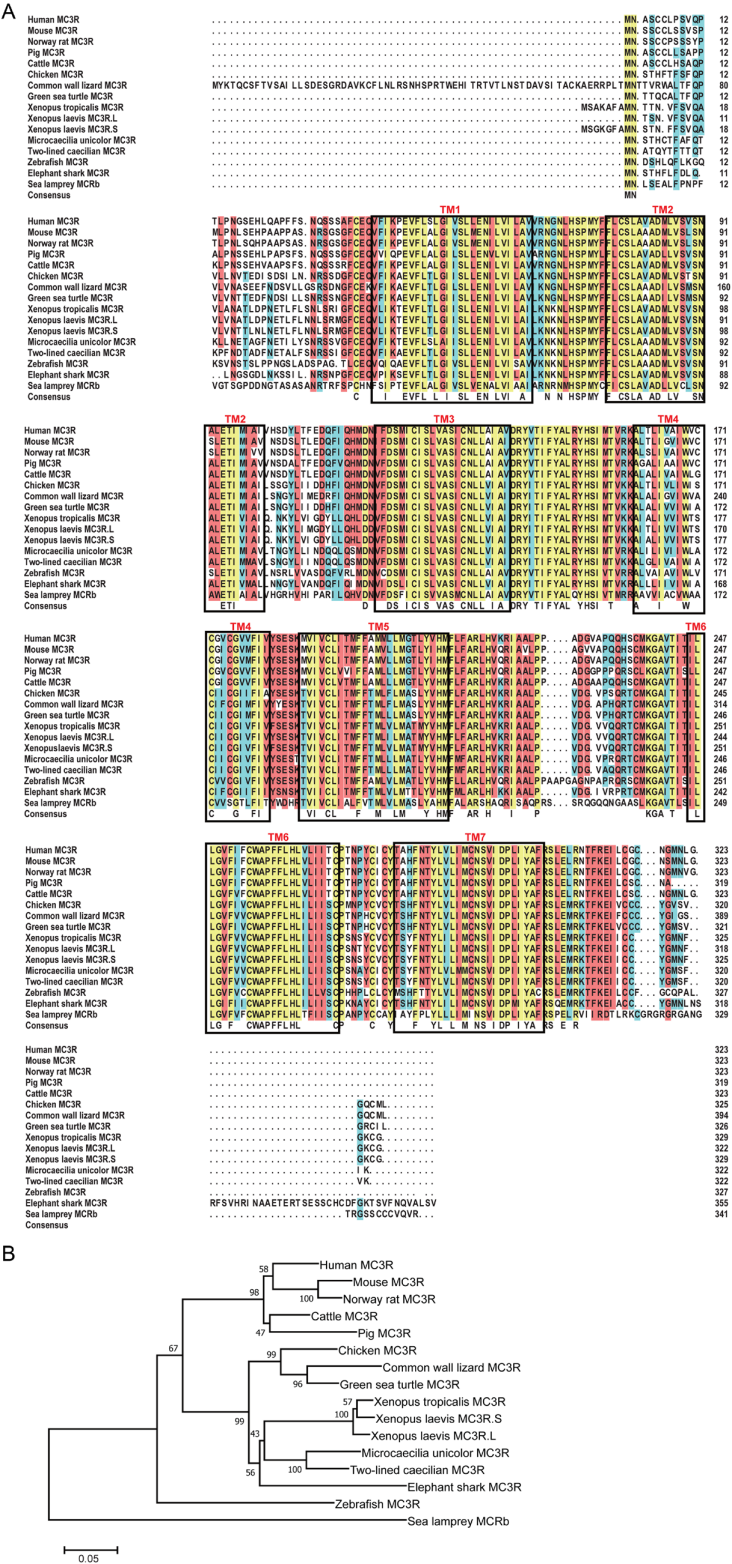
Protein alignment and phylogenetic analysis of *Xenopus laevis* MC3Rs. (A) Sequence alignments of xlMC3Rs (*Xenopus laevis* MC3R.L, XP_018090440.1; *Xenopus laevis* MC3R.S, XP_018093682.1) and other MC3Rs from human (NP_063941.3), mouse (NP_032587.1), Norway rat (NP_001020441.3), pig (NP_001116609.1), cattle (XP_010809919.1), chicken (XP_004947293.1), common wall lizard (XP_028591076.1), green sea turtle (XP_007059824.1), *Xenopus tropicalis* (XP_002935436.1), *Microcaecilia unicolor* (XP_030069319.1), two-lined caecilian (XP_029467773.1), zebrafish (NP_851303.2), elephant shark (XP_007883784.1), and sea lamprey (ABB36647.1). The blue, red, and yellow represent a homology over 50%, 75%, and 100%, respectively. (B) Dendrogram of MC3Rs was generated by the NJ analysis with Molecular Evolutionary Genetics Analysis (MEGA) software.

**Figure 2:**
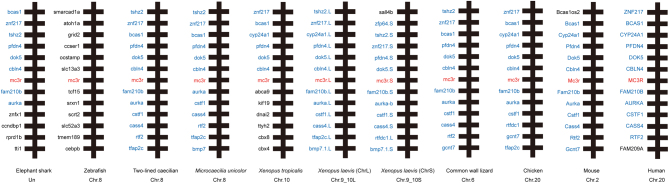
Synteny analysis of *Xenopus laevis* MC3Rs. Synteny mapping of MC3Rs with *Callorhinchus milii* (elephant shark), *Danio rerio* (zebrafish), *Rhinatrema bivittatum* (two-lined caecilian), *Microcaecilia unicolor*, *Xenopus tropicalis*, *Xenopus laevis*, *Podarcis muralis* (common wall lizard), *Gallus gallus* (chicken), *Mus musculus* (house mouse), and *Homo sapiens* (human). Genes in blue represent positional genomic conservatism during evolution among multiple species.

**Figure 3:**
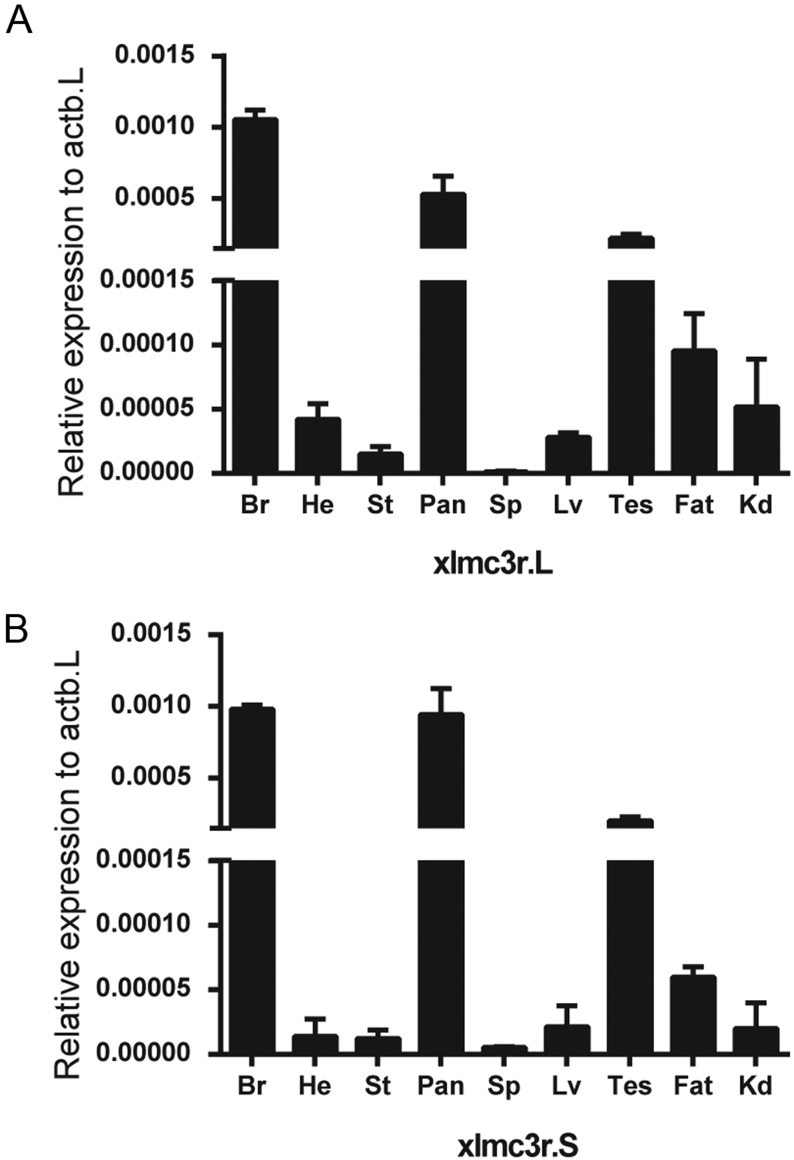
mRNA expression of xlmc3r.L/S. Expression profiles of xlmc3r.L (A) and xlmc3r.S (B) in multiple tissues from an adult male *Xenopus laevis*. The relative expression was normalized to the housekeeping gene xlactb.L. Data were plotted as mean ± s.e.m. of three independent experiments. Br, brain; He, heart; St, stomach; Pan, pancreas; Sp, spleen; Lv, liver; Tes, testis; fat; Kd, kidney.

The discovery of ancestor MCRs had been reported in the genomes of lamprey and hagfish, indicating that the melanocortin system existed only in chordates (27, 28). To analyze the evolutionary conservation of xlMC3Rs, we selected several chordates including five mammals (human, mouse, Norway rat, pig, and cattle), one bird (chicken), two reptiles (common wall lizard and green sea turtle), four amphibians (*Xenopustropicalis*, *Xenopuslaevis*, *Microcaecilia unicolor*, and two-lined caecilian), two fishes (zebrafish and elephant shark), and one cyclostomata (sea lamprey) and performed the protein sequence alignment (Fig. 1A). xlMC3R.L showed 96.3% identity with xlMC3R.S. Moreover, the transmembrane domains of xlMC3R.L and xlMC3R.S also exhibited high similarity in all chordates. To further identify the evolutionary relationship of MC3R between *Xenopuslaevis* and other chordates, we constructed the phylogenetic tree of all MC3Rs. As predicted, amphibians involving *Xenopustropicalis*, *Xenopuslaevis*, *Microcaecilia unicolor,* and two-lined caecilian were clustered into a distinct clade in the dendrogram (Fig. 1B). Next, we examined the genomic regions of MC3R in elephant shark, zebrafish, two-lined caecilian, *Microcaecilia unicolor*, *Xenopustropicalis*, *Xenopuslaevis*, common wall lizard, chicken, mouse, and human and illustrated adjacent gene orders surrounding xlMC3R.L and xlMC3R.S for synteny analysis (Fig. 2). The upstream and downstream genes of xlMC3Rs, including cbln4, dok5, pfdn4, aurka, cstf1, and cass4, were in great concordance with amphibians and mammalian species. We collected nine tissues and explored the mRNA expression of xlmc3r.L/S by quantitative RT-PCR (Fig. 3). Like mammalian distribution patterns, both xlmc3r.L and xlmc3r.S showed high expression in the brain region (29). Notably, mrap2 is also distributed in the brain in *Xenopustropicalis* (xt) and *Xenopuslaevis*, with the difference that mrap was relatively less expressed in this tissue of *Xenopuslaevis* (18, 30). Furthermore, both xlmc3rs and xlmraps were mainly expressed in the pancreas and testis (Fig. 3) (30). Collectively, both phylogeny and synteny of xlmc3rs verified compelling evolutionary conservation, while the expression profile of xlmc3rs in the brain probably suggested its participation in the regulation of physiological energy homeostasis.

### Co-localization and protein interaction of xlMC3Rs and xlMRAPs in vitro

**Figure 4:**
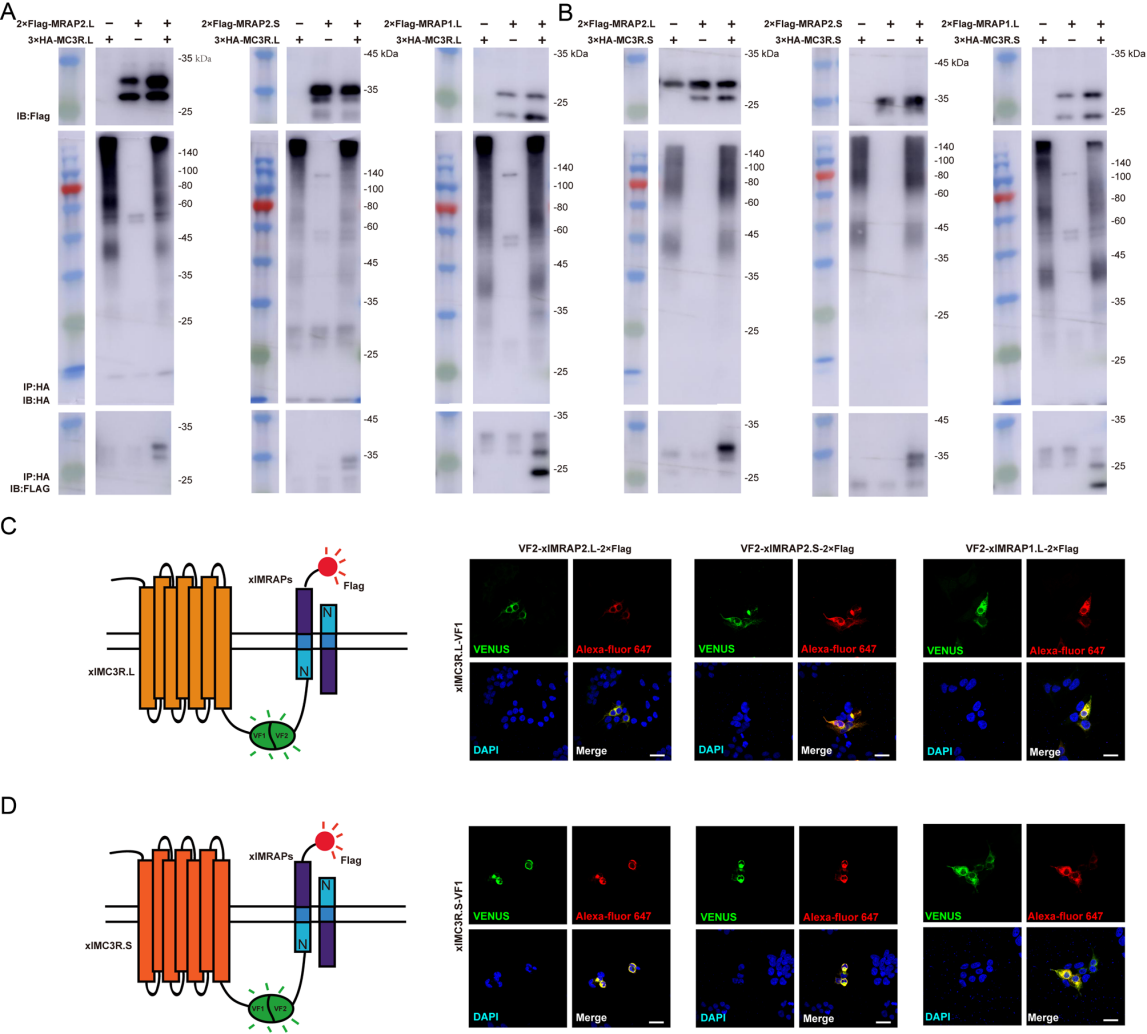
Protein interaction of xlMRAPs and xlMC3Rs* in vitro*. (A) Co-immunoprecipitation of the 3×HA-xlMC3R.L and 2×Flag-xlMRAPs. (B) Co-immunoprecipitation of 3×HA-xlMC3R.S and 2×Flag-xlMRAPs. The bimolecular fluorescence complementation assay showed the co-localizations of (C) xlMC3R.L or (D) xlMC3R.S and xlMRAPs. Venus fluorescence imaging (green) and 2-Flag (red) exhibited protein complex of xlMRAP1.L (or xlMRAP2.L/S) and xlMC3Rs. Nuclei were in blue (DAPI). Scale bar = 10 μm.

To identify the protein interaction of xlMC3Rs with xlMRAPs, we first co-transfected 3×HA-xlMC3Rs and 2×Flag-xlMRAPs into HEK293T cells and detected a direct physical protein complex* in vitro* (Fig. 4A and  B). Moreover, we examined the co-localization of xlMC3Rs and xlMRAPs in HEK293T cells. A bimolecular fluorescence complementation study was recruited to pinpoint the xlMC3Rs–xlMRAPs complex. VF1 tagged xlMC3Rs in C-terminal while N-terminally VF2 and C-terminally 2×Flag were designed for xlMRAPs. Fluorescence generated by the protein complex was localized in intracellular compartments and plasma membrane (Fig. 4C and  D). Overall, Co-IP and dual fluorescent assay verified that the xlMRAPs could interact with xlMC3Rs* in vitro*.

### Pharmacological characterization of xlMRAPs on xlMC3Rs signaling

**Figure 5:**
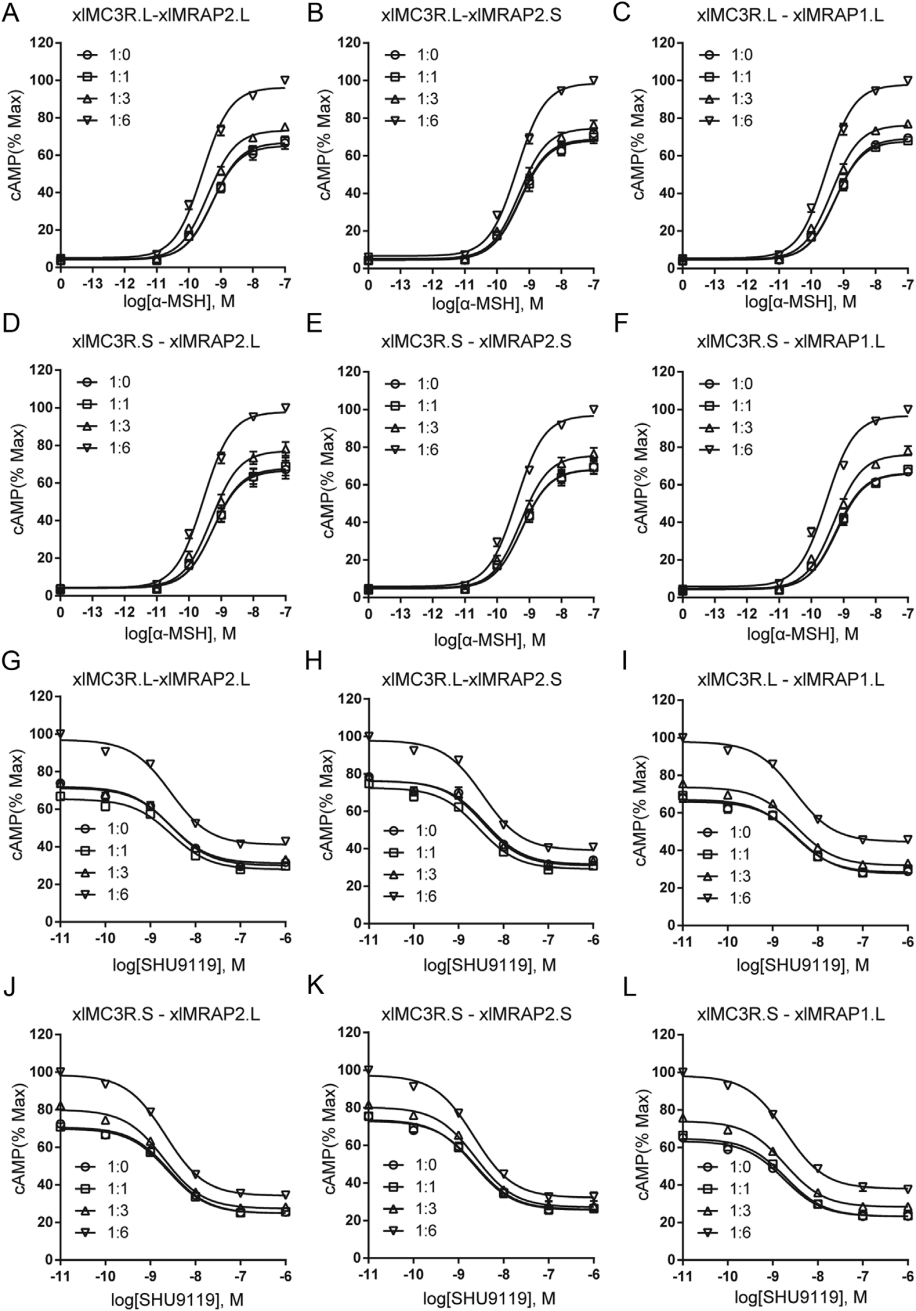
Modulation of xlMC3R.L/S signaling by xlMRAPs. (A, B, C, D, E, and F) Dose–response curves of agonist (α-MSH) (0 M, 10^−11^ to 10^−^7 M) stimulated cAMP production of xlMC3R.L with different ratio of (A) xlMRAP2.L, (B) xlMRAP2.S, and (C) xlMRAP1.L. Ligand stimulation of xlMC3R.S was also modulated by (D) xlMRAP2.L, (E) xlMRAP2.S, and (F) xlMRAP1.L. All data of ligand stimulation were normalized to the maxima of 1:0, 1:1, 1:3, and 1:6 curves in the ligand stimulation assay. Data were plotted as the mean ± s.e.m. of three independent experiments performed in triplicate. (G, H, I, J, K, and L) The antagonistic ability of SHU9119 (10^−11^ to 10^−6^ M) in the presence of α-MSH (EC_80_) induced the alteration of xlMC3R.L signaling with different amounts of (G) xlMRAP2.L, (H) xlMRAP2.S, and (I) xlMRAP1.L. Antagonistic ability of SHU9119 (10^−11^ to 10^−6^ M) in the presence of α-MSH (EC_80_) induced the alteration of xlMC3R.S signaling was also regulated by (J) xlMRAP2.L, (K) xlMRAP2.S, and (L) xlMRAP1.L. All data of antagonistic ability were normalized to the maxima of 1:0, 1:1, 1:3, and 1:6 curves. Data were plotted as the mean ± s.e.m. of three independent experiments performed in triplicate.

It is known that POMC-derived peptides activated MCRs signaling, and MRAPs could modulate the expression and response of all MCRs (12, 13, 31, 32). MC3R in various species was regulated by MRAP and MRAP2 inconsistently when stimulated with agonists (12, 14, 15, 16, 17). Functional differences among species spurred us to explore the pharmacological modulation of xlMRAP2.L/S (or xlMRAP1.L) on xlMC3R.L/S signaling. Our results showed that all three accessory proteins did not dramatically influence the constitutive activities of xlMC3R.L/S. Both xlMC3R.L and xlMC3R.S showed robust α-MSH-stimulated cAMP signaling in the presence of the higher amount of xlMRAPs (Fig. 5A, B, C, D, E, and  F). Next, we tested the inhibitory efficacy of SHU9119, a synthetic melanocortin antagonist for MC3R and MC4R, on xlMC3Rs signaling EC80 dose of α-MSH (33, 34). An opposite pharmacological action showed that SHU9119 inhibited the activity of xlMC3R.L/S. We found that xlMRAP1 and xlMRAP2s dose dependently inhibited the SHU9119-induced reduction of MC3R activity (Fig. 5G, H, I, J, K, and L). The reduction range of each group went from 60 to 40%. In addition, EC50 in each group was not observed to be significantly changed in the presence of xlMRAP1 or xlMRAP2s, indicating that xlMRAP1 and xlMRAP2s did not affect the sensitivity of xlMC3R to SHU9119. Altogether, with luciferase reporter assay, we observed the dose-dependent increase of cAMP production of xlMC3Rs with xlMRAPs.

### Influence of xlMRAPs on surface expression of xlMC3Rs

**Figure 6:**
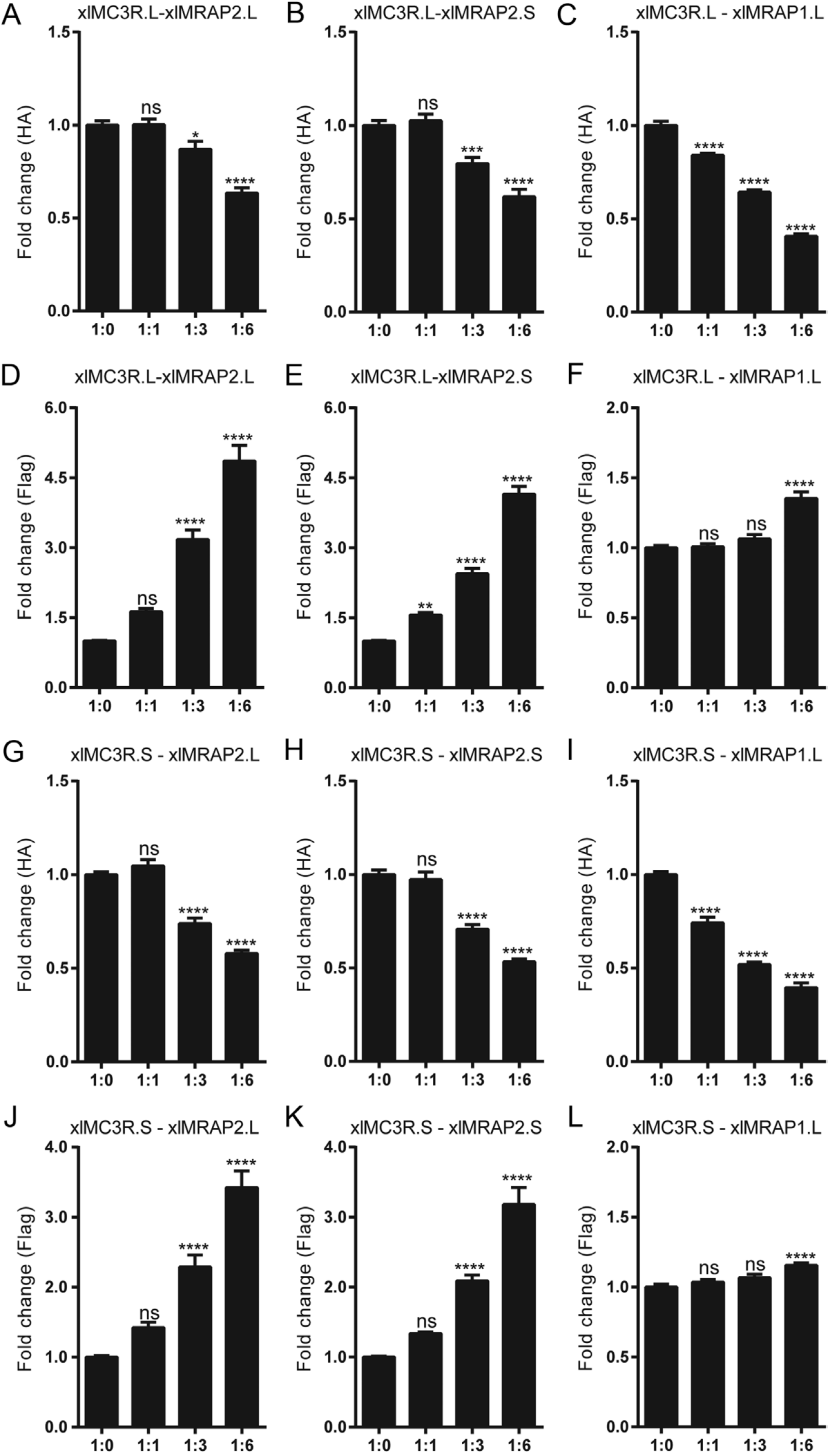
Alteration of the surface expression of xlMC3R.L and xlMC3R.S by xlMRAPs at ratio of 1:0, 1:1, 1:3, and 1:6. Surface expression of the N-terminally 3×HA tagged xlMC3R.L in the presence of (A) the N-terminally 2×Flag tagged xlMRAP2.L, (B) the N-terminally 2×Flag tagged xlMRAP2.S, or (C) the N-terminally 2×Flag tagged xlMRAP1.L. Whole expression of the N-terminally 2×Flag tagged xlMRAP2.L (D and J), the N-terminally 2×Flag tagged xlMRAP2.S (E and K), and the N-terminally 2×Flag tagged xlMRAP1.L (F and L). Surface expression of the N-terminally 3×HA tagged xlMC3R.S in the presence of (G) the N-terminally 2×Flag tagged xlMRAP2.L, (H) the N-terminally 2×Flag tagged xlMRAP2.S, or (I) the N-terminally 2×Flag tagged xlMRAP1.L. Each receptor/accessory protein expression level was shown as fold difference compared to HEK293T cells expressing 3×HA-xlMC3R alone. Data were plotted as the mean ± s.e.m. of three independent experiments performed in triplicate, not significant [ns], ∗*P* < 0.05, ∗∗*P* < 0.01, ∗∗∗*P* < 0.001, ∗∗∗∗*P* < 0.0001 one-way ANOVA.

Next, we detected whether the altered activity of xlMC3Rs was related to the surface expression of xlMC3R.L/S* in vitro*. To achieve this goal, 3×HA-xlMC3Rs and 2×Flag-xlMRAPs were co-transfected at four different ratios (1:0, 1:1, 1:3, and 1:6). Then, we performed ELISA to measure the surface expression level of xlMC3R.L/S. As shown in Fig. 6, accompanied by an increment of the whole xlMRAP2.L (or xlMRAP2.S) (Fig. 6D, E, J, and  K), the surface expression of xlMC3R.L/S decreased significantly (Fig. 6A, B, G, and  H). The surface expression of xlMC3R.L/S was all significantly reduced by approximately 40% at a 1:6 ratio of xlMRAP2s compared with the control group. A similar change of xlMC3R.L/S surface expression level was also observed in the presence of xlMRAP1.L (Fig. 6C, F, I, and L). However, we found that the effect of xlMRAP1 seemed to be greater than xlMRAP2s on xlMC3R.L/S surface expression, as MRAP1 decreased the surface expression of xlMC3R.L/S by 60% in the 1:6 ratio compared to the control group. Overall, these results showed that both xlMRAP2s and xlMRAP1.L inhibited the surface expression of xlMC3Rs.

## Discussion

*Xenopuslaevis* is an ideal model system to explore genomic duplication and functional divergence because of its allotetraploidization. In this research, we have verified the high evolutionary conservation of xlMC3Rs. The phylogenetic tree clearly showed that xlMC3R in S chromosome showed the closest relative with the diploid *Xenopustropicalis* among organisms from cyclostomata to mammalian (Fig. 1B). In multiple protein alignment and synteny analysis, we elucidated that xlMC3R.L/S is conversed in amino acid sequence (Fig. 1A) and in genomic regions among amphibians (Fig. 2). The high mRNA expression of both xlmc3r.L and xlmc3r.S in the brain (Fig. 3), where previous reports had demonstrated (16, 29, 35, 36), probably indicated similar physiological functions among distinct species. We also clarified the interaction between xlMC3Rs and xlMRAPs by Co-IP assay and the protein complex located on the plasma membrane by bimolecular fluorescence complementation analysis *in vitro* (Fig. 4).

Earlier studies showed MCRs co-interacted with MRAP or MRAP2 and significantly influenced the trafficking of MCRs from the endoplasmic reticulum to the cell surface in vertebrates (8, 11, 12, 37). We further explored the response of xlMC3R.L/S to natural ligand modulated by xlMRAPs and measured the receptor-mediated cAMP production stimulated by α-MSH or antagonized by SHU9119, respectively (Fig. 5). The activity of xlMC3R.L/S could be stimulated by α-MSH and inhibited by SHU9119, consistent with previous reports in vertebrates (17, 32, 38). Differently, little effect was seen on the constitutive activity of xlMC3R.L/S in the presence of increasing concentrations of xlMRAPs. However, we observed a remarkable influence on xlMC3R.L/S cAMP signaling under the circumstance of an increasing dose of xlMRAPs proteins. Four ratios of xlMRAPs were transfected to verify whether the activity was due to the changeable surface expression of xlMC3Rs (Fig. 6). Surprisingly, xlMRAPs inhibited the surface level of xlMC3Rs considerably. Both surface xlMC3R.L and xlMC3R.S were easily influenced by a higher percentage of xlMRAP2.L/S (Fig. 6A, B, G and  H). Similarly, MRAP2 also increased the signaling of MC3R when activated by α-MSH and inhibited its expression at the cell surface in mouse studies (14, 15). Unlikely, co-transfected MRAP or MRAP2 with MC3R had no significant influence on the surface expression of MC3R in human (12). Other studies also showed a functional divergence of MRAP or MRAP2 on modulating MC3R surface expression. In chickens, compared to the significant alteration by MRAP2, the α-MSH-stimulated cAMP level of MC3R remained unchanged by MRAP1 (16). In channel catfish, the presence of MRAP2 inhibited MC3R responsiveness to α-MSH (17). In *Xenopustropicalis*, a higher ratio of MRAP or MRAP2 also increased the α-MSH-stimulated cAMP signaling of MC3R. The same as xlMRAP2s, xtMRAP2 inhibited the surface expression of xtMC3R. However, unlike xlMRAP1.L, the xtMRAP1 significantly increased the cell surface expression of xtMC3R (18). These findings demonstrated that xlMRAPs exerted a similar influence on xlMC3R.L/S activation and surface expression.

In summary, we performed the first comprehensive analysis in evolutionary conservation and functional divergence of two homologous MC3Rs in the allotetraploid frog *Xenopuslaevis in vitro*. The metamorphosis, evolutionary-induced polyploidization, and the huge size of the African clawed frog may be closely related to the regulation of energy metabolism. Distinct pharmacological profiles* in vitro* with different gene combinations of MC3R and MRAPs in *Xenopuslaevis* and *Xenopustropicalis* spurred us to further focus on the evolutionary conservation and variability of MCRs functions* in vivo* in amphibians.

## Declaration of interest

The authors declare that there is no conflict of interest that could be perceived as prejudicing the impartiality of the research reported.

## Funding

The work was supported by grants from National Key Research and Development Program of China (Grant No. 2017YFA0103902 and 2019YFA0111400); the National Natural Science Foundation of China http://dx.doi.org/10.13039/501100001809 (Grant No. 31771283 and 31771608), the Fundamental Research Funds for the Central Universities http://dx.doi.org/10.13039/501100012226 (No. 22120190210), Innovative Research Team of High-level Local Universities in Shanghai (Grant No. SSMU-ZDCX20180700), and a Key Laboratory Program of the Education Commission of Shanghai Municipality (Grant No. ZDSYS14005).
